# Identification of Risk Factors Associated with Obesity and Overweight—A Machine Learning Overview

**DOI:** 10.3390/s20092734

**Published:** 2020-05-11

**Authors:** Ayan Chatterjee, Martin W. Gerdes, Santiago G. Martinez

**Affiliations:** 1 Department of Information and Communication Technology, Centre for e-Health, University of Agder, 4604 Kristiansand, Norway; martin.gerdes@uia.no; 2 Department of Health and Nursing Science, Centre for e-Health, University of Agder, 4604 Kristiansand, Norway; santiago.martinez@uia.no

**Keywords:** obesity, overweight, BMI, machine learning, classification, regression, obesity, lifestyle diseases, normal distribution, gradient descent, hypothesis test, data visualization, python, Sklearn, eCoach, deep learning, Prisma, model performance, discrimination, calibration, monitoring, sensor data

## Abstract

Social determining factors such as the adverse influence of globalization, supermarket growth, fast unplanned urbanization, sedentary lifestyle, economy, and social position slowly develop behavioral risk factors in humans. Behavioral risk factors such as unhealthy habits, improper diet, and physical inactivity lead to physiological risks, and “obesity/overweight” is one of the consequences. “Obesity and overweight” are one of the major lifestyle diseases that leads to other health conditions, such as cardiovascular diseases (CVDs), chronic obstructive pulmonary disease (COPD), cancer, diabetes type II, hypertension, and depression. It is not restricted within the age and socio-economic background of human beings. The “World Health Organization” (WHO) has anticipated that 30% of global death will be caused by lifestyle diseases by 2030 and it can be prevented with the appropriate identification of associated risk factors and behavioral intervention plans. Health behavior change should be given priority to avoid life-threatening damages. The primary purpose of this study is not to present a risk prediction model but to provide a review of various machine learning (ML) methods and their execution using available sample health data in a public repository related to lifestyle diseases, such as obesity, CVDs, and diabetes type II. In this study, we targeted people, both male and female, in the age group of >20 and <60, excluding pregnancy and genetic factors. This paper qualifies as a tutorial article on how to use different ML methods to identify potential risk factors of obesity/overweight. Although institutions such as “Center for Disease Control and Prevention (CDC)” and “National Institute for Clinical Excellence (NICE)” guidelines work to understand the cause and consequences of overweight/obesity, we aimed to utilize the potential of data science to assess the correlated risk factors of obesity/overweight after analyzing the existing datasets available in “Kaggle” and “University of California, Irvine (UCI) database”, and to check how the potential risk factors are changing with the change in body-energy imbalance with data-visualization techniques and regression analysis. Analyzing existing obesity/overweight related data using machine learning algorithms did not produce any brand-new risk factors, but it helped us to understand: (a) how are identified risk factors related to weight change and how do we visualize it? (b) what will be the nature of the data (potential monitorable risk factors) to be collected over time to develop our intended eCoach system for the promotion of a healthy lifestyle targeting “obesity and overweight” as a study case in the future? (c) why have we used the existing “Kaggle” and “UCI” datasets for our preliminary study? (d) which classification and regression models are performing better with a corresponding limited volume of the dataset following performance metrics?

## 1. Introduction

More than one-third of the adult population in the United States is obese and this is linked to certain factors, such as physical inactivity, improper diet, family history, and the environment [1]. As reported by “The GBD 2015 Obesity Collaborators” in 2015, a total of 107.7 million children and 603.7 million adults were obese [2]. After analyzing data from 68.5 million people from 195 countries between 1990 and 2015, the research team concluded that the burden of “obesity and overweight” is related to high body-mass index (BMI), age, and gender. With the number of obese people doubling in two decades (from 1.3 million people obese globally in 1980 to double in 2008), unhealthy habits (such as consumption of tobacco and alcoholic beverages), unhealthy diet (such as energy drinks, consumption of excess salt and sugar, intake of high saturated fat, and discretionary foods), and physical inactivity are the major pillars of “obesity and overweight”. In 2016, more than 1.9 billion adults (39%) aged eighteen years and older were overweight, and of these, over 650 million (13%) were obese. In 2016, more than 340 million children and teenagers aged five to nineteen were overweight or obese, and in 2018, 40 million children under the age of five were overweight or obese. The universal predominance of “obesity and overweight” nearly tripled between 1975 and 2016. Juvenile obesity is linked to a higher chance of obesity, untimely death, and infirmity in adulthood [3,4,5,6]. The chronic conditions associated with “obesity and overweight” are considered as health care and social burdens. According to the latest study conducted by the “National Health and Nutrition Examination Survey (NHANES, 2007–2012)” on aggregated data (2007–2008, 2009–2010, and 2011–2012) collected from 15,208 adults with an age >=25, excluding pregnancy (n = 125) and incorrect noisy data (n = 827), a significant correlation was observed between sex, age, race, or ethnicity with “obesity and overweight” [1]. Potential risk factors related to obesity/overweight may vary in children under age five, adolescents, adults, older people, and pregnant women.

The consequences of “Obesity and overweight”, which continues to be the foremost public health anxiety, increases the risk of the other four primary lifestyle diseases, such as cardiovascular diseases (CVD), cancers, diabetes (type II), and chronic lung diseases (chronic obstructive pulmonary disease (COPD), asthma). The burden of these diseases is extremely high among lower-income countries and populations. A total of 63% (36 million) of global death occurred in 2008 due to lifestyle diseases or non-communicable diseases (NCDs). Additionally, 80% of the 36 million dead people belonged to low- and middle-income classes, 13% were from high-income classes, and 29% of the total NCD deaths occurred below the age of sixty years. An increase of 10 million deaths annually on average due to NCDs has been observed from selected literature study. In 2016, the number increased to 56.9 million (71%), and by 2030, it is predicted to achieve 75%, with 88.5% death in developed countries and 65% death in developing countries. The risk for the stated lifestyle diseases increases with “body mass index” (BMI) in direct proportion. BMI is a number calculated by “Weight/Height^2^” and is used to assess body composition [3,4,5,6,7]. However, BMI is rather a bad indicator of percent of body fat, as BMI does not capture information on the mass of fat in different body sites and is highly dependent on age [8]. In 2012, as identified by the Institute of Medicine, population-based obesity prevention strategies, such as physical activity, healthy diet, models of healthy social rules, and context-based and tailored recommendations by setting have the potential to combat “obesity and overweight” [9]. Thus, health behavior change should be given precedence to circumvent severe damages. 

An electronic coaching (“eCoaching”) system can empower people to manage a healthy lifestyle with early risk predictions and appropriate individualized recommendations. To develop an intelligent eCoach system for automated, personalized, contextual, and behavioral recommendations to achieve personal wellness goals, addressing obesity as a study case, we propose to (a) identify associated health risk factors, (b) perform data collection from identified controlled trials, (c) analyze the data, and (d) perform a predictive analysis with machine learning algorithms for future health risk predictions and behavioral interventions [10,11]. 

In this tutorial of ML models to identify the risk factors of overweight and obesity, we reviewed the performance of different machine learning algorithms (regression and classification) on existing datasets available in “Kaggle” and “UCI” so that we could create a list of risk factors associated with obesity/overweight with an appropriate quantitative analysis. The obtained result at the end of the study helped us to decide which risk factors health and wellness data would be collected on for our future research work—“eCoach behavioral interventions for obesity and overweight”. 

A comparative performance analysis of different classification algorithms helped to hypothesize which model to use under which circumstances, such as data volume, binary class, or multi-class classification. “Childhood and elderly obesity”, “obesity and genetic profiling”, “obesity and pregnancy”, “nutrition”, “recommendation generation and goal evaluation”, and “robotic interaction” are beyond the scope of this paper. Our primary focus was obesity/overweight in adults. In this paper, we used the term “eCoaching” [10,11], which is our future research focus for behavioral intervention for the promotion of a healthy lifestyle targeting “obesity and overweight” as a study case. “eCoaching” as such is also not in the scope of this paper. 

The main contributions of this paper are as follows:

(1) Identifying a set of risk factors associated with obesity/overweight following different established statistical methods on health datasets available in “Kaggle” [12] and “UCI” [13];
(2) Understanding how the identified risk factors are correlated to weight change with regression analysis and data visualization techniques;
(3) Reviewing various machine learning (ML) models for the classification and regression of the same selected datasets.

The remainder of the paper is structured as follows. In Section 2, we summarize the methodology for the study selection. In Section 3, we describe the related works along with a brief elaboration on how we searched, selected, and reviewed scientific literature in this context. Section 4 presents the methodology utilized related to the data selection, data analysis, statistical analysis, ML model training and testing, ML model evaluation, model reuse, and the assessment of body composition in adults. In Section 5, we discuss our analysis and findings. Section 6 ends with the conclusions of the paper. This study can be considered as a tutorial on using ML models to identify the risk factors of overweight and obesity, because there is no prior hypothesis on any specific risk factors. To reproduce the results as discussed in Section 5, codebase has been uploaded in GitHub as mentioned in Section “Supplementary Materials”.

## 2. Methodology for Study Selection

**Figure 1:**
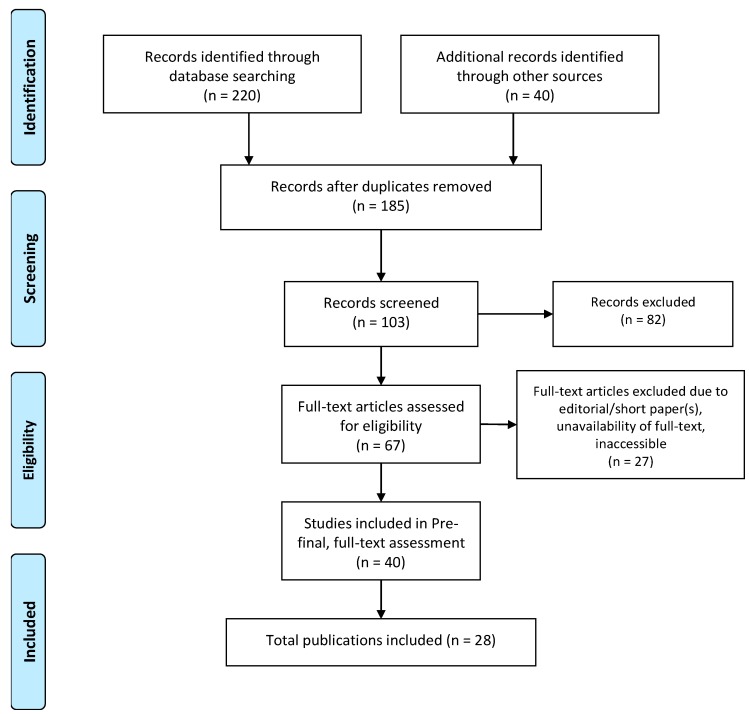
Prisma flowchart for the article selection process [16].

To complete this study, we reviewed scientific literature published between 2012 and 2019 and retrieved from “Google Scholar”, “PubMed”, “Scopus”, “Science citation index (SCI)”, “IEEE Xplore”, “SpringerLink”, and “MDPI”. A Prisma evidence-based framework was used for the systematic review and meta-analyses [14,15]. The tools used to make the searching and selection of articles successful were “EndNote”, “DOAJ”, “Sherpa/Romeo”, and “Microsoft Excel”. We aimed to include articles that described the utilization of different machine learning and deep learning algorithms on “obesity and overweight” and related datasets. Searched articles were categorized into the following four categories: quantitative, qualitative, editorial, and book. Searching was based on appropriate keywords, as mentioned in the “Keyword” section. We reviewed the abstracts and conclusions of about 67 papers, and in the final phase we selected 40 articles for full-text reading. Twenty-eight out of the 40 articles are cited in the reference section, and for reference management, we used “EndNote” software. A total of 10 online web articles from the WHO, Centers for Disease Control and Prevention (CDC), Sklearn, python, and National Institute for Clinical Excellence (NICE) were analyzed and cited in the reference section. We excluded papers - not written in English; articles related to child obesity, genetics, nutrition, robotic interaction, and pregnancy; short papers, editorial papers, or papers without full text; articles published beyond the searched timeframe (2012–2019), articles not indexed in “Google Scholar”; articles with the most similar contents or duplicate papers; and inaccessible articles. The epidemiological study design for this paper is described in Table 1. The complete flowchart of the selection process, following - identification, screening, eligibility, and inclusion is depicted in Figure 1. 

The study of epidemiology is related to how often lifestyle diseases (obesity/overweight) occur in different groups of people, why, and a potential list of risks. Epidemiological information is used here to plan and evaluate strategies to prevent obesity/overweight in the future and as a guide to the supervision of patients in whom the disease has already developed [17].

## 3. Related Work

“Obesity and overweight” remains as a significant public health problem not only in the USA but also in other countries for the last ten to fifteen years. It has prevailed among pre-school students and childbearing-age women at a low rate but is increasing among school students rapidly and scores high in adults, mainly in the group of girls or women with less education or schooling. In developed countries, it occurs mostly in vulnerable groups of the economically weak population, and the opposite occurs in less developed societies as household nutrition transition and underweight can coexist with weight increase. Obesity tends to decline with increasing income. In developed countries, women are suffering almost double when compared to men in the lower socioeconomic group [18]. Projects have been conducted by different research groups on “obesity-related risk predictions with machine learning and deep learning approaches” to generate useful regression and classification models.

Singh et al. [19] evaluated different multivariate regression methods and multilayer perceptron (MLP) feed-forward neural network models on the dataset obtained from a millennium cohort study (MCS) with over 90% accuracy to predict teenager BMI from previous BMI values. Twenty neurons in the hidden layer resulted in the lowest mean absolute error (MAE), with a mean training time of 1.63 s and a regularization factor of 0.9.

Bassam et al. [20] performed a study on data obtained from the Kuwait Health Network (KHN) to build prognostic models to predict the future risk of diabetes (type II) using machine learning algorithms (logistic regression, k-nearest neighbor (KNN), support vector machine (SVM)) with a five-fold cross-validation technique. The study included age, sex, body mass index (BMI), pre-existing hypertension, family history of hypertension, and diabetes (type II) as baseline non-invasive parameters. As a result, KNN outperformed the other models, with area under the ROC (receiver operating characteristic) curve (AUC)values of 0.83, 0.82, and 0.79 for 3-, 5-, and 7-year prediction limits.

Meghana et al. [21] used “auto-sklearn”, an automatic machine learning (AutoML) library for developing classifiers of CVDs. They experimented on both the heart UCI dataset and a cardiovascular disease dataset consisting of 70,000 records of patients and, as a result, AutoML outperformed traditional machine learning classifiers.

Seyla et al. [22] studied how to classify obesity from dietary and physical activity patterns using machine learning classification algorithms and, as a result, support vector machine (SVM) outperformed other classifiers.

Jindal et al. [23] performed ensemble machine learning approaches for obesity prediction based on the key determinants—age, height, weight, and “BMI”. The ensemble model utilized Random Forest (RF), generalized linear model, and partial least square, with a prediction accuracy of 89.68%. Grabner at al. performed a study on “National Health and Nutrition Examination Survey (NHANES)”, “National Health Interview Survey (NHIS)”, and “Behavioral Risk Factor Surveillance System (BRFSS)” datasets from the 1970s to 2008 to analyze the trend of BMI in the USA over time and across race, gender, socioeconomic background, and status (SES). It was observed that SES–BMI gradients were steadily more significant for women than for men. 

Zheng et al. [24] used binary logistic regression, improved decision tree (IDT), weighted k-nearest neighbor (KNN), and artificial neural network (ANN) on nine health-related behaviors from the 2015 Youth Risk Behavior Surveillance System (YRBSS) for the state of Tennessee in their study to predict obesity in high school students by focusing on both risk and protective factors. The result showed that the IDT model achieved an 80.23% accuracy and 90.74% specificity, the weighted KNN model achieved an 88.82% accuracy and 93.44% specificity, and the ANN model achieved an 84.22% accuracy and 99.46% specificity in the classification problem.

Dunstan et al. [25] used three non-linear machine learning algorithms—SVM, Random Forest (RF), and Extreme Gradient Boosting (XGB) to predict obesity incidence at the country level, based on countrywide sales of a small subset of food and beverage classes. The study predicted that baked goods and flours, followed by cheese and sweet carbonated drinks, were the most pertinent food categories to predict obesity.

DeGregory et al. [26] suggested in their literature review of “machine learning in obesity” that smart wearable wireless sensors, electronic medical health records, smartphone apps, and insurance data are rich sources of obesity-related data and are quite promising to treat and prevent obesity/overweight. Machine learning algorithms do have the potential to describe, classify, and predict obesity-related risks and consequences. They reviewed various machine learning methods, such as linear and logistic regression, artificial neural networks, deep learning, decision tree analysis, cluster analysis, principal component analysis (PCA), network science, and topological data analysis with the strengths and limitations of each method on the National Health and Nutrition Examination Survey to demonstrate the methodology, utility, and outcomes. 

Golino et al. [27] used a machine learning technique, namely, a classification tree, to investigate the prediction of increased blood pressure by body mass index (BMI), waist (WC) and hip circumference (HC), and waist–hip ratio (WHR) on 400 college students from 16-63 years of age (56.3% women). The model outperformed the traditional logistic regression model in terms of predictive power. The model presented a sensitivity of 80.86% and specificity of 81.22% in the training set and, respectively, 45.65% and 65.15% in the test sample for women and a sensitivity of 72% and specificity of 86.25% in the training set and, respectively, 58.38% and 69.70% in the test set for men.

The relationship between body fat and anthropometry is quite popular in obesity calculation. In the following three different ways, anthropometric measurements can be conducted: (a) BMI, (b) waist circumference, and (c) hip circumference to body fat. Pleuss et al. [28] conducted a machine learning-based study in 3D image processing to obtain hundreds of anthropometric measurements within seconds after analyzing the images obtained from a 3D scanner.

Maharana et al. [29] used the convolutional neural network (CNN) approach on approximately 150,000 high-resolution satellite images from Google Static Maps application program interface (API) to check associations between the built environment and obesity; their developed regression model concluded that obesity varied across studies and geographical contexts. The cross-sectional study was conducted on 1695 census areas in six cities, and the data on adult obesity prevalence were obtained from the Centers for Disease Control and Prevention’s 500 Cities project.

Obesity/overweight is a consequence of an energy imbalance in our body. Therefore, a proper diet is also essential along with a physical activity to balance calories intake and consumption. Pouladzadeh et al. [30] proposed a deep learning (CNN)-based solution with 10,000 high-resolution food images for system training that would run on the smartphones as an application and would have the capability to take a picture of the food and calculate the amount of calorie intake automatically.

Machine learning and deep learning are natural extensions to conventional statistical methods. It has become an essential tool for the modern healthcare system. Whether an algorithm is high or low on the machine learning or deep learning continuum, the best rational methods must be utilized to ensure that the result is robust and valid. It is true in healthcare because these algorithms can affect the lives of millions of people [2]. From the related work, we identified a list of machine learning (ML) and deep learning (DL) models and risk factors related to obesity/overweight, as described in Table 2. In Section 4, we only executed machine learning models on the available sample health data in a public repository. Execution of the deep learning models will be performed in a future study.

## 4. Methods

**Figure 2:**
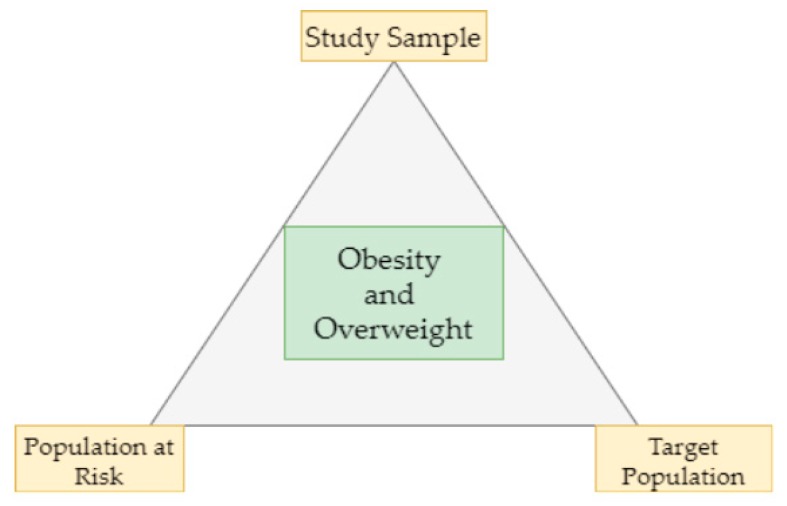
The focused epidemiological study triangle [33].

We utilized the established ML models in our research to perform a statistical analysis on available public datasets in “Kaggle”, [12] and “UCI” [13] to study the correlation between the identified risk factors and weight change. Subsequently, we evaluated the performance of different machine learning models for classification and regression. The overall process includes data collection, data pre-processing, statistical analysis and data visualization, algorithm selection for classification and feature predictions, model training and testing, model evaluation, and model reuse [31,32]. In this study, we focused on three things—the population at risk, the study sample, and the target population (Figure 2). We studied different samples of data and the corresponding target population, as described in Section 4.1, on the population at risk in the age group of >20 and <60, excluding pregnancy and genetic factors. In this study, we have not predicted any brand-new risk factors.

### 4.1. Data Collection

We found in the selected literature study that BMI (height, weight); sex; age; environment; blood pressure; and behavioral risks, such as physical inactivity, improper diet, body energy imbalance, and habit are the foremost risk factors of obesity/overweight. Obesity/overweight gradually develops CVDs and diabetes type II in humans with deliberate economic decline. We hypothesize that obesity/overweight correlates with CVDs and diabetes and that some common risk factors exist, such as age, sex, cholesterol, lipid profile, sugar level, blood pressure, and family history.

In this study, we performed a regression analysis to visualize the trend of change in age, tobacco consumption, sweet beverages, economic condition, fast food, sleeping pattern, diet, blood pressure, blood glucose, lipid profile, adiposity, exercise, and family history in relation to obesity/overweight/weight change/CVDs/diabetes type II in the sample population, excluding genetic factors and pregnancy. Currently, we have no collected data that combine all the intended risk factors in a single dataset, eligible for this study. In our future research related to “behavioral interventions through eCoaching for obesity”, we have a plan to collect data related to identified risk factors from south Norway in both males and females with an age group of >20 and <60, excluding pregnancy and genetic factors. Thus, for our current study, we focused on existing health datasets. From the review, we identified three potential public reliable sources of data, namely “Kaggle” [12], “UCI” [13], and “Physio Net” [34]. Our primary target was to find existing health-related data (obesity/overweight/CVDs/diabetes type II) from a reputed and reliable machine learning data repository; we found our required data in “Kaggle” and “UCI” with proper references. Most of the “UCI” data are available in “Kaggle”. After a proper background verification of the data, we selected 5 sets of data, as summarized in Table 3. The explanation and source of the corresponding data are available in the “Kaggle” and “UCI” web portals.

The data obtained from both “Kaggle” and “UCI” are not from the same sources and the same target population. Their data volume is also different, but they contain most of the identified potential risk factors. Hence, combining the data into a single source is merely difficult. It might result in a very small single set of data after the removal of all unnecessary heterogeneous features, and the resulting dataset might be inappropriate for machine learning model training with cross-validation. That is the reason we processed the individual datasets separately with the identified risk-features obtained from the literature study. Our focused population age was >20 and <60, without pregnancy and genetic factors. A short description of the data is provided in Table 4. The data from different sources have added a provision to find if there are any more risk factors associated with it. The selected data are classified into three categories—(a) obesity, (b) diabetes type II, and (c) CVDs. The identified key features, as described in Table 4, were used for the machine learning model training for both regression and classification.

### 4.2. Data Processing

The collected data are categorized among two groups—continuous and categorical. The accumulated data in this research are labeled. We have used supervised machine learning models (classification and regression) for training and testing the accuracy. Several selected datasets are small, some are noisy, and the remaining contain a good volume of data to train the supervised machine learning model. Data mining was included to filter the data samples from each of the datasets and to discard samples containing outliers. Data mining involves pattern discovery, the calculation of feature association (and correlation), feature selection, classification, clustering, and outlier analysis. 

During data cleaning, we removed data that were incomplete, beyond the age >20 and <60, and features such as pregnancy, having children/number of children. Data processing incorporates three steps, as stated below [24,25,30,31]:

- Data preprocessing includes data integration, the removal of noisy data that are incomplete and inconsistent, data normalization and feature scaling, encoding of the categorical data, feature selection after correlation analysis, and split data for training and testing a machine learning model.
- Training of a machine learning model and testing its accuracy with a k-fold cross validation.
- Data postprocessing includes pattern evaluation, pattern selection, pattern interpretation, and pattern visualization.

In this experiment, we have used python 3.x language libraries for the data processing, as described in Table 5. We set up a “Python” environment using an anaconda distribution and used spyder IDE for developing the python-based “data science” applications. 

### 4.3. Statistical Analysis 

Statistical analysis of the selected datasets involves the following methods, as stated in Table 6. According to the central limit theorem, when a bunch of random numbers is added together, it produces a normal distribution. The normal distribution can be described entirely by the two parameters µ (mean) and σ (standard deviation). As always, the mean is the center of the distribution, and the standard deviation is the measure of the variation around the mean. Let random variable “X” follow the normal or gaussian distribution (bell curve) if the probability of the density function of “X” is presented by $f\left(x\right)=1/\sigma \surd 2\mu \;e^{-1/2\left(\left(x-\mu \right)/\sigma \right)2},\;-\infty <x<+\infty$ and the area under the normal curve is 1% or 100%. 

The probability of normal distribution can be calculated through the standard normal distribution “Z” (|Z|=|(X−μ)/σ|). The Z-score transformation is a linear transformation with µ = 0 and σ = 1, is used for feature scaling. A normality test is used to check whether a distribution is gaussian. The normal distribution is symmetric about µ. This leaves that the area to the left of µ is equal to the area to the right of µ. 

Hypothesis testing is a statistical method that is used in achieving statistical decisions using trial data. A hypothesis test estimates two mutually exclusive statements about a population to ascertain which statement is supported by the trial data. The critical parameter of hypothesis testing is the null hypothesis (**H_0_**) that tells us there is nothing different or significant about the data. On the contrary, the alternative hypothesis (**Ha**) directly contradicts H_0_. The confidence factor or value of significance (α) is used to decide whether to accept or reject an **H_0_**. The value of α is usually kept as 0.05% or 5%, as 100% accuracy is impossible to achieve whether accepting or rejecting **H_0_**. Popular widely used hypothesis testing methods, a short description, and the required sample size are demonstrated in Table 7. A hypothesis test can be either a one-tailed test or a two-tailed test. For each of the testing methods, the resulting probability value (*P-value*) is compared with “α” to accept or reject a null hypothesis. However, it may carry type-I error (false positive) or type-II error (false negative) [31,32,39]. 

Example:

**H_0_**: Sample looks “Gaussian”.

**H_a_**: Sample does not look like “Gaussian” and α = 5% or 0.05.

“Shapiro-Wil”k, “D” Agostino’s K^2, and “Anderson-Darling” test calculate the P-value to decide if a sample looks like gaussian (P-value > α = 0.05) or not (P-value < α = 0.05). Covariance (COV(x,y)) is a property of a function to retain its form when its variables are linearly transformed. It helps to measure the correlation (r_xy_) that measures the strength of the linear relationship between two variables.

(1) corr(x,y)=COV(x,y)/(σx∗σy),  where−1<r<+1.

“Sign” shows the direction of the relationship among two variables x and y. Table 8 shows the meaning of different |r| values. If two variables are strongly correlated, it is recommended to select any one of them during feature selection. Pearson’s correlation coefficient is used to summarize the strength of the linear relationship between two variables in normal distribution and Spearman’s correlation is used to calculate the non-linear relationship between two variables [31,32,39]. 

A quantile analysis divides the distribution into four parts—min, Q1 (25%), median, Q3 (75%), and max. The interquartile range (IQR = Q3 − Q1) is a measure of data dispersion and used to check if data (X) are outliers or not. Data (X) are outliers if:

(2) X < Q1−1.5∗(IQR) or X > Q3+1.5∗(IQR).

### 4.4. Model Training and Testing

In this study, we have selected machine learning algorithms for the classification and regression analysis, as described in Table 9 and explained in Section 5.

The steps used to train and test a machine learning model are described below:

- Load data.
- Data pre-processing:
  ⚬ remove missing values from the loaded data;
  ⚬ encode categorical features;
  ⚬ check distribution of data and features;
  ⚬ remove data from outliers;
  ⚬ remove data redundancy;
  ⚬ correlation analysis among features and feature scaling if required. We compared the correlation between features and removed one of two features that had a correlation higher than 0.9;
  ⚬ column/feature selection based on the p-value with the help of “regressor_OLS”;
  ⚬ visualize the distribution of selected features;
  ⚬ shuffle the data.
- Split data for training and testing (80:20) with some random state.
- Machine learning model selection, as described in Table 6, based on regression or classification problem statement and building the model.
- K-fold cross validation on data (in our study, K = 5).
- Perform a prediction.
- Evaluate the model performance with metrics, as described in Section 4.5, following discrimination and calibration-based performance measures.
- Perform model tuning with a “grid search” parameter optimization technique.

**Note:** **a.** Selection of learning rate (α): if too small then it slows the convergence in the gradient descent (GD), and if too large then it slows the convergence in GD or the GD may diverge. 

**b.** Let “m” training samples have “n” features. If there are too many features (m <= n), then delete some features or use regularization with the regularization factor ‘λ’.

**c.** If ‘λ’ is too large then the algorithm fails to eliminate overfitting, or even sometimes underfits and the GD fails to converge. ‘λ’ (∞) increases to lead a high bias and decreases to lead a high variance.

**d.** Underfitting results in a high bias and overfitting leads to a high variance. 

**e.** If a learning algorithm is suffering from a high bias, more training data will not help much. If a learning algorithm is suffering from high variance, more training data is likely to help.

**f.** C = (1/λ) = line separation effect in SVM: large “C” leads to a lower bias and high variance, small ‘C’ leads to a higher bias and low variance.

**g.** Gradient descent follows the convex optimization technique with upper bound (L) and lower bound (µ) on the curvature f:

(3) $$µI_{d\;}\leq \;\nabla^{2}f\left(x\right)\leq LI_{d\;},\;where\;\nabla^{2}f\left(x\right)\;is\;the\;Hessian,\;µ>0\;\;and\;L=Lipschitz\;continious.$$

### 4.5. Model Evaluation

The developed machine learning models for classification and regression are evaluated with the following metrics: [28,31,32,40]

- Classification metrics: accuracy score, classification report, and confusion matrix.
- Regression metrics: mean absolute error (MAE), mean squared error (MSE), and R^2^-score.
- Calibration: Goodness-of-fit statistics with a Brier score metric for binary classification. The Brier score is a metric which is a combination of the calibration loss and refinement loss. Calibration loss is the mean squared deviation from the empirical probabilities derived from the slope of the ‘Receiver Operating Characteristic (ROC)’ segments. Refinement loss is the expected optimal loss as measured by the area under the optimal cost curve [41].

The classification report includes precision, recall, and F1-score. A confusion matrix is a table with two dimensions, “actual” and “predicted”, and both the dimensions have “true positives (TP)”, “true negatives (TN)”, “false positives (FP)”, “false negatives (FN)”.

- TP—both actual class and predicted class of data point is 1.
- TN—both actual class and predicted class of data point is 0.
- FP—actual class of data point is 0 and predicted class of data point is 1.
- FN—actual class of data point is 1 and predicted class of data point is 0.

Formulas for calculating the classification metrics are stated as below:

(4) $$\begin{array}{c} Accuracy=\frac{\left(TP+TN\right)}{TP+FP+FN+TN}\;,\;Precision\;\left(P\right)=\frac{TP}{\left(TP+FN\right)},\\ Recall\;\left(R\right)\;or\;Sensitivity\;\left(S\right)=\frac{TP}{TP+FN},\\ Specificity=\left(1-Sensitivity\right)=\frac{TN}{TN+FP\;\;},\;F1\;score=2*\frac{PR}{P+R}. \end{array}$$

Accuracy tells us how close a measured value is to the real one. Precision determines how close a measured value is to the real one. Recall or sensitivity defines the total number of positives (actual) returned by the machine learning model. 

MAE is the easiest error metric used in the regression problem following the formula:

(5) $$MAE=\frac{1}{n}\;\sum |Y-\hat{Y}|,\;where\;Y=actual\;value\;and\;\hat{Y}=predicted\;value.$$

MSE squares the difference of actual and predicted output before adding them all instead of using the absolute value following the formula:

(6) $$MSE=\frac{1}{n}\;\sum (Y-{\hat{Y)}}^{2}\;,\;where\;Y=actual\;value\;and\;\hat{Y}=predicted\;value.$$

An R^2^ regression metric has been used for an explanatory purpose to provide an indication of the fitness in the predicted output values to the actual output values. It is calculated with a formula with the numerator as the MSE and the denominator as the variance in Y values.

(7) $$R^{2}=1-\frac{\frac{1}{n}\;\sum_{i=1}^{n}(Y-{\hat{Y)}}^{2}}{\left(\frac{1}{n}\;\sum_{i=1}^{n}(Y-{\bar{Y}}^{2}\right)}\;,\;where=actual\;value,\;\hat{Y}=predicted\;value,\;and\;\bar{Y}=mean\;value.$$

Calibration implies the measure of the agreement between observations and predictions. It is a post-processing technique to enhance the error distribution of a predictive ML model. It helps to understand how the resulting errors are distributed and how well the probability estimations are made. Though many ML techniques are good in overall results, they might have a bad evaluation of the distribution of error. To develop a calibrated classification model, we followed two steps—probability prediction, and prediction of calibration with a reliability diagram/calibration plot. A reliability diagram/calibration plot describes how well the forecast probabilities are calibrated with a comparative frequency of what was observed (Y-axis) versus the predicted probability frequency (X-axis). The better calibrated or more reliable a forecast, the closer the points might appear along the main diagonal from the bottom left to the top right of the plot. The position of the points or the curve relative to the diagonal might help to interpret the probabilities. For example, (a) below the diagonal, the model has over-forecast as the probabilities are too large; (b) above the diagonal, the model has under-forecast as the probabilities are too small. An “S-shaped” curve expresses pessimistic tendencies, over-forecasting low probabilities and under-forecasting high probabilities. There are two calibration techniques, as follows—sigmoid or Platt’s scaling, and isotonic scaling/regression [40,42,43,44]. We used the “Brier Score” [41] for the binary class classification as a metric which indicates the smaller the Brier score, the better the calibration. To calibrate the binary scores/probabilities, we reduced the multiclass problems to a binary classification problem. Then, we compared the relativity curves of different classification problems and followed by choosing the best model based on the minimum Brier score for both the “sigmoid” and “isotonic” methods. A well-calibrated classifier is a probabilistic classifier for which the output of the “predict_proba” [41] method can be directly inferred as a confidence level. Platte scaling is very efficient when the distortion/bias in the predicted probabilities is sigmoid (“S”) shaped, else, we can use isotonic regression. For large training sets, isotonic regression is useful.

(8) $$Brier\;Score=\frac{1}{N}\;\sum_{i=1}^{N}{\left(f\left(t\right)-o\left(t\right)\right)}^{2},$$

where *f*(*t*) = the probability that was forecast and *o*(*t*) = the actual outcome of the event at instance *t*.

Note: the logic to convert a multiclass problem to a binary classification problem is as follows: if (BMI ≤ 24.9) then the predicted class will be “class-0” or “class-1”.

### 4.6. Model Store and Reuse

We saved our final trained machine learning model in a file and restored it to use it again either by comparing the model with other models or by testing the model on new or updated data. The process of storing the model is called serialization, and restoring the model is called deserialization. It can be done in two ways, as described in Table 10. The pickled model can be stored in the database for distributed access.

### 4.7. Assessment of Body Composition

The assessment of body composition is performed with two popular techniques—(a) BMI and (b) waist–hip ratio. A waist–hip ratio of > 0.85 and 1.00 are associated with a greater than average risk in women and men, respectively. Here, the BMI has been used to categorize different weight groups in adults of twenty years or older for both men and women [26]:

- Weak: BMI < 18.5.
- Normal weight: BMI is 18.5 to 24.9.
- Overweight: BMI is 25 to 29.9.
- Obesity class I: BMI >= 30.0 and BMI <= 34.9.
- Obesity class II: BMI >= 35.0 and BMI <= 39.9.
- Obesity class III: BMI >= 40.0.

In this study, we considered “obesity class II” and “obesity class III” as extreme obesity. This study excluded pregnant women and genetic factors. 

## 5. Results and Discussions

**Figure 3:**
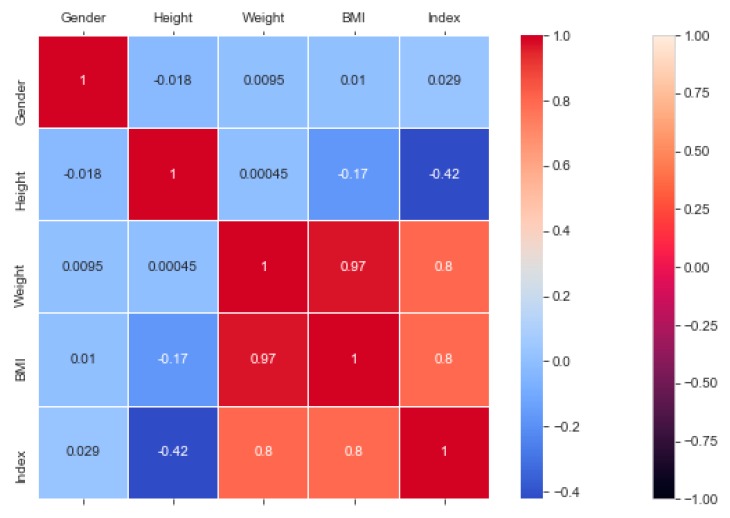
Correlation heatmap and classification accuracy of ML models to classify “BMI” data.

**Figure 4:**
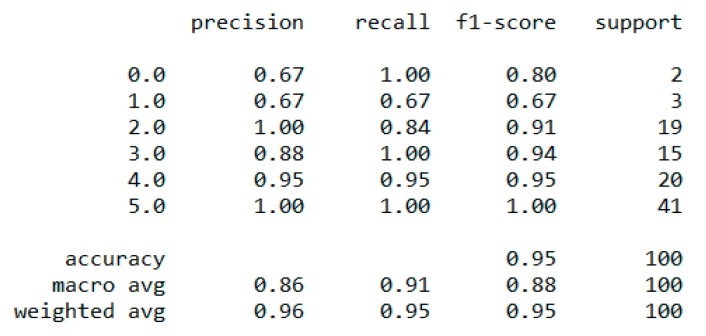
Performance metric of “SVM” classification with 5-fold cross validation.

The “BMI” dataset has 500 records with four features—“gender”, “height”, “weight”, and “index”. The dataset has no missing or incomplete data. The “index” determines whether a person is extremely weak (0), weak (1), normal weight (2), overweight (3), obese (4), or extremely obese (5). We added the extra feature “BMI” (weight/height2) to the dataset in pre-processing, and later it was removed during the model training due to high correlation. The remaining features are chosen based on the hypothesis testing (p-value). The correlation in Figure 3 exhibits a strong correlation between “BMI” and “index” (obesity-determining class), and it is appropriate in accordance with Section 4.7. We used the dataset for a multiclass classification to group people according to their body composition. For the multiclass classification, we used “SVM” with linear and radial basis function (RBF) kernel, “Naïve Bayes” (gaussian and current), “Decision Tree (DT)” (gini and gain), “RF” (estimators 50 and 100), and “KNN” (neighbor 2 and 6) models, but “SVM” with linear kernel provided the best classification, with an accuracy = 0.95, MSE = 0.08, R^2^ = 0.96, and MAE = 0.06 with the 5-fold cross-validation technique. The best parameters of SVM are {’C’: 0.01, ’gamma’: 0.001, ‘kernel’: linear}, with a score of 95% following the grid search method. The resultant performance metrics of “SVM” are depicted in Figure 4. 

**Figure 5:**
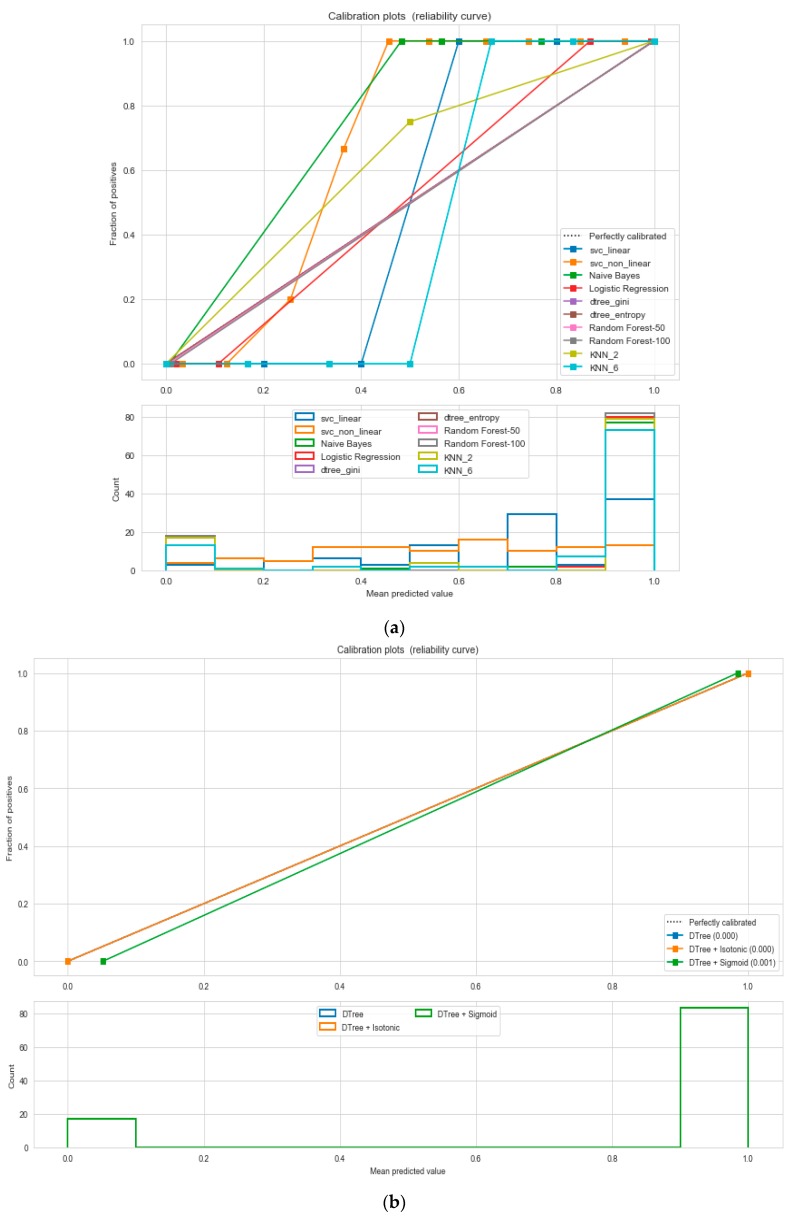
(**a**) Reliability curve to classify the “BMI” data with different ML classifiers. (**b**) Reliability curve to classify the “BMI” data with “Calibrated Decision Tree”.

For the calibrated classification technique, we converted the multiclass “BMI” dataset to a binary classification problem by adding an extra prediction feature class “Risk” following the logic that if (extremely weak, weak, normal weight) then “Risk” = 0, else 1. Both the uncalibrated “SVM” and “Decision Tree” classifiers performed the best in binary classification. In contrast, the calibrated “Decision Tree” classifier performed the best, as depicted in the Figure 5, following the “isotonic” calibration method with a Brier score = 0.000. It shows that the uncalibrated and calibrated “Decision Tree” classifiers give equal performance.

**Figure 6:**
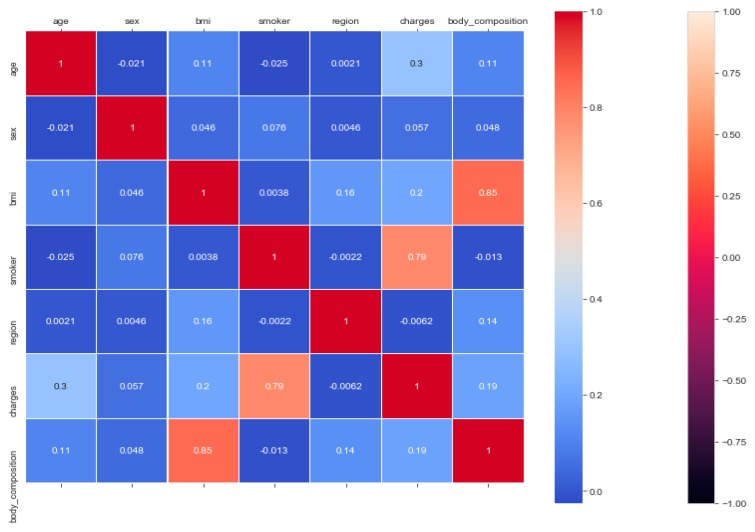
Correlation heatmap and classification accuracy of ML models to classify “Insurance” data.

**Figure 7:**
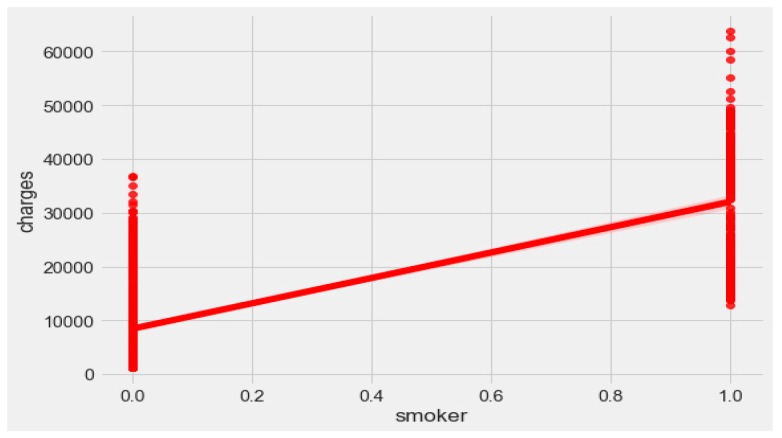
Relationship between “smoker” and “charges”.

The “insurance” dataset has 1338 records and seven features—“age”, “sex”, “BMI”, “children”, “smoker”, “region”, and “charges”. During data pre-processing, we included records with an age >20 and <60 and excluded the feature “children”. It resulted in 1058 records. Then, we added the extra feature “body_composition” based on the “BMI” feature, and the feature classified the records among four classes—underweight (0), normal weight (1), overweight (2), and obese (3). We encoded the categorical features such as sex, smoker, and region. We found a strong correlation between “smoking” and “charge”, with |r| = 0.79, as depicted in Figure 6 and Figure 7. Smoking is one of the most frequently negative health behaviors in humans. Negative health behavior has a great impact on weight change, as found in the literature study. If the charge of the insurance increases, then it might lead to an adverse effect on personal or family financial planning, and it is one of the consequences of lifestyle diseases as predicted by WHO 4]. Thus, excess smoking does not only create a powerful negative impact on health but also creates a passive negative impact on economic position. We used insurance data for both the classification and regression analysis.

**Figure 8:**
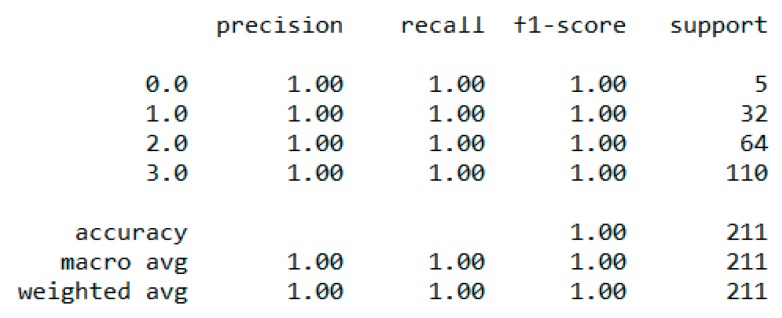
Performance metric of “Decision Tree” classification with 5-fold cross validation.

In classification, we used “body_composition” as a predicted feature, and the “Decision Tree” model performed the best with a 99.64% accuracy as depicted in Figure 8. 

**Figure 9:**
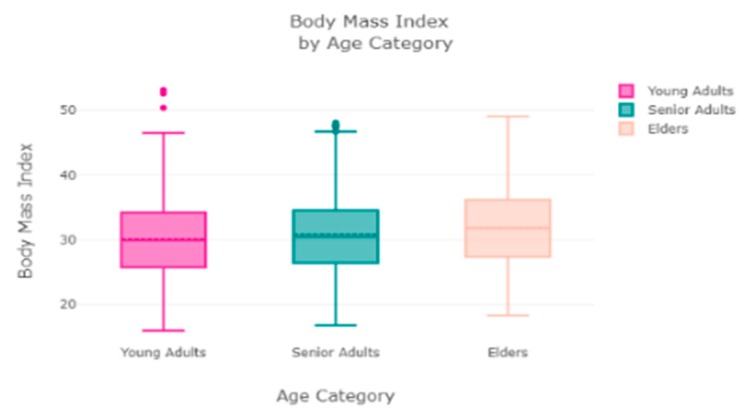
Relationship between “age category” and “BMI”.

**Figure 10:**
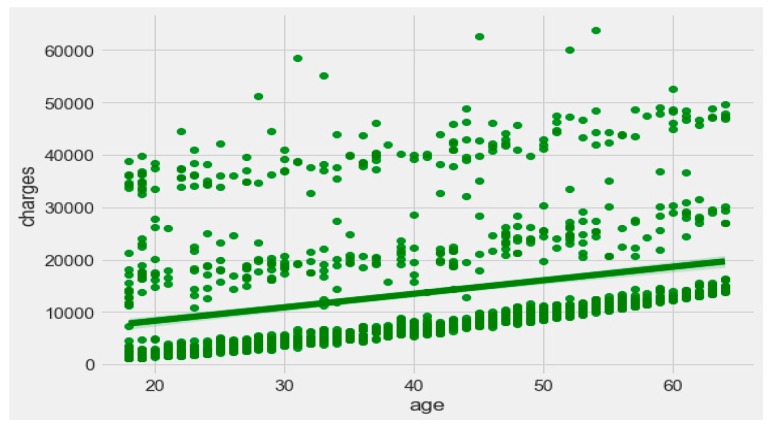
Relationship between “age category” and “charges”.

**Figure 11:**
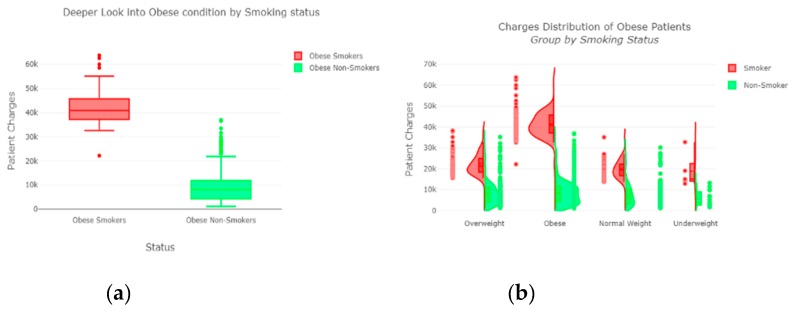
(**a**) Obese condition by smoking status; (**b**) distribution of obese patient group by smoking status.

The finest parameters of the “Decision Tree” classifier are {best criterion: entropy, best max_depth: 24, best number of components: 6, min_samples_leaf=1, min_samples_split=2, and splitter=’best’}, following the grid search method. For the regression, we used “charges” as a predicted feature and performed the hypothesis testing with “ANOVA” results to retain Ha= {a significant change between the three age categories (young adults, senior adults, elders) with “BMI”}, with a P-value of 0.001, 0.060, and 0.000, respectively. The boxplot analysis in Figure 9 exhibits that “BMI” increases with “age”, and average “BMI”s for each of the groups are in the obesity range, which is a risk. Therefore, the body composition changes with increasing age both in males and females, and this is a risk to humans. The pattern of the data demonstrates that the charge of the insurance increases with increasing age, as depicted in Figure 10. From Figure 9, it is evident that BMI increases with age. Hence, with a transitive relation, it is evident that the charge of the insurance increases with increasing BMI. The change in insurance charge with smoking condition and age is depicted in Figure 11.

**Figure 12:**
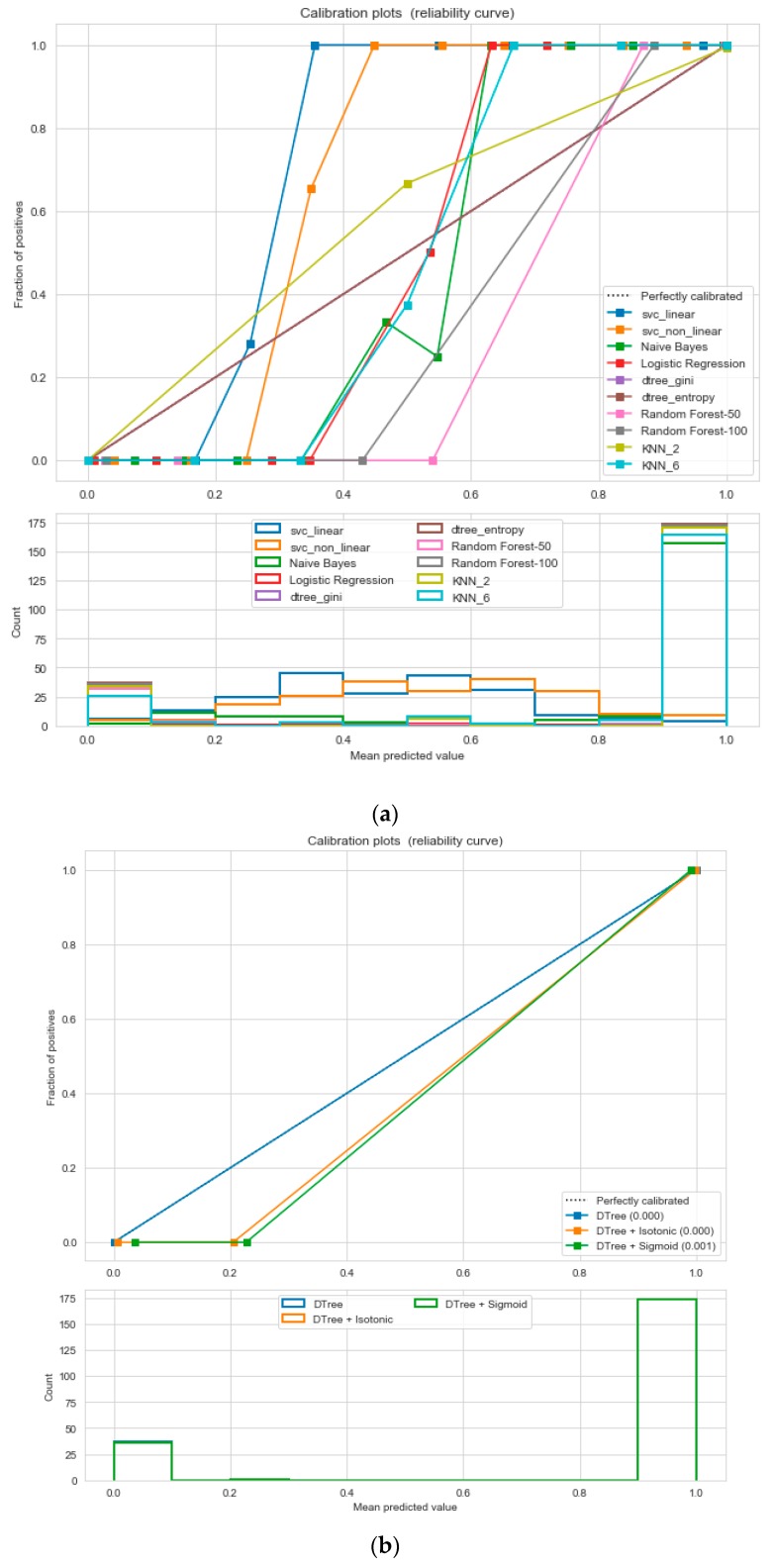
(**a**) Reliability curve to classify “Insurance” data with different ML classifiers. (**b**) Reliability curve to classify “Insurance” data with the “Calibrated Decision Tree”.

For the calibrated classification, we converted the multiclass “insurance” dataset to a binary classification problem following the logic that if (underweight, normal weight) then “body_composition” = 0, else 1. The uncalibrated “SVM”, “Decision Tree”, and “Random Forest” classifiers performed the best in the binary classification. On the contrary, the calibrated “DecisionTree” classifier outperformed the other classifiers, as depicted in Figure 12, following the “isotonic” calibration method with a Brier score = 0.000, where the Brier scores for “SVM”, and “RF” were 0.216 and 0.001, respectively. It demonstrates that the uncalibrated and calibrated “Decision Tree” classifiers give a similar performance. After a performance comparison of the regression algorithms, we found that “Random Forest” performed the best, with an 81% accuracy. The finest parameters for the “Random Forest” regressor were {’bootstrap’: True, ’max_depth’: 50, ’max_features’: 5, ’min_samples_leaf’: 6, ’min_samples_split’: 8, ’n_estimators’: 100} following the grid search method.

**Figure 13:**
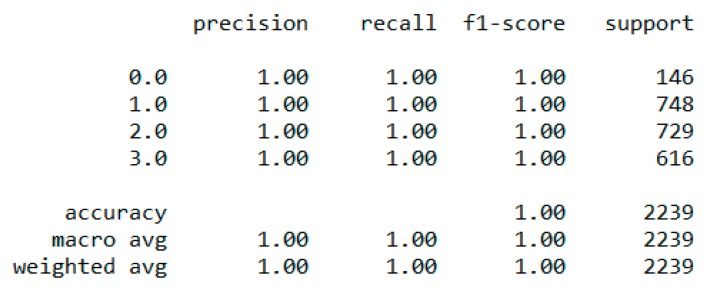
Performance metric of the “Decision Tree” classification with a 5-fold cross validation.

The “Eating-health-module” dataset has 11,212 records and many features, but we included the following important features after the correlation study—“erbmi”, “eusoda”, “eusnap”, “euincome2”, “eugenhth”, “erincome”, “eudietsoda”, “euffyday”, “eufdsit”, “eufastfdfrq”, “ertseat”, “eudrink2, “eueat”, “euexfreq”, “euexercise”, “eufastfd”, “eumeat”, “eumilk”, “eustores”, “eustreason”, “euwic”. During the data pre-processing, we included records with a value > 1 and excluded features such as “erincome” due to high correlation [37]. It resulted in 11,192 records and 21 columns. Then, we added an extra feature, “body_composition”, based on the “erbmi” feature, and the feature classified the records among four classes—underweight (0), normal weight (1), overweight (2), and obese (3). We processed the data with ML classification algorithms to classify the records based on the “body_composition” feature, and the “Decision Tree” classifier performed the best with a 99.7% accuracy, as depicted in Figure 13. The finest parameters of the “Decision Tree” classifier were {best criterion: entropy, best max_depth: 36, best number of components: 13, min_samples_leaf=1, min_samples_split=2, and splitter=’best’} following the grid search method. The regression analysis of the selected feature revealed that sweet beverages, economic condition, fast food, sleeping, meat and milk consumption, a drinking habit, and exercise have a sharp impact on growing obesity in humans.

**Figure 14:**
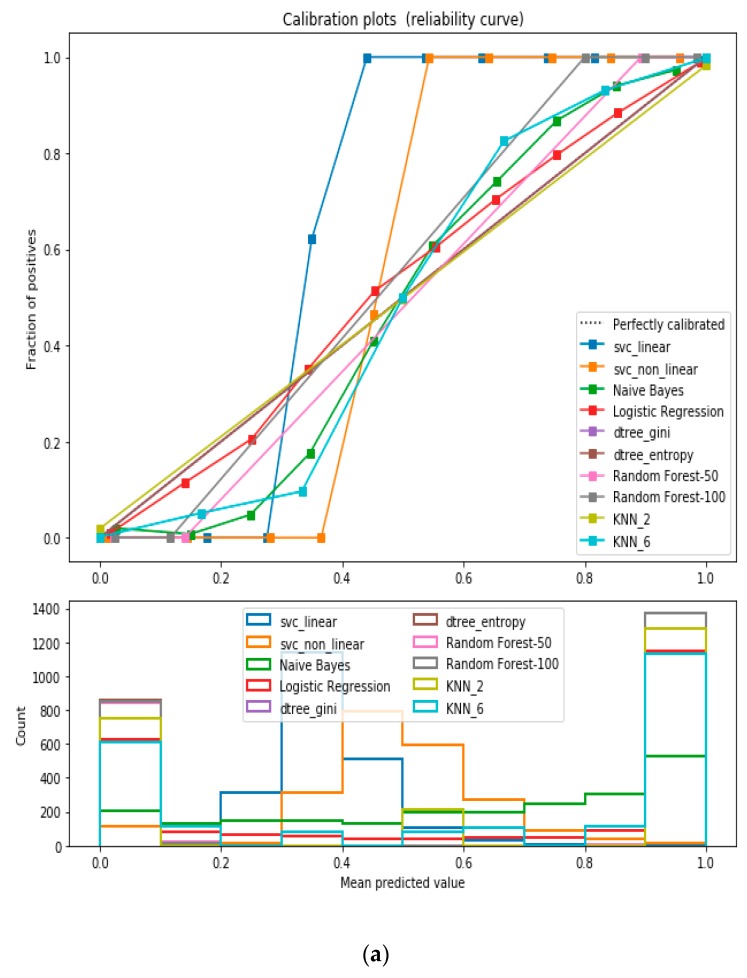

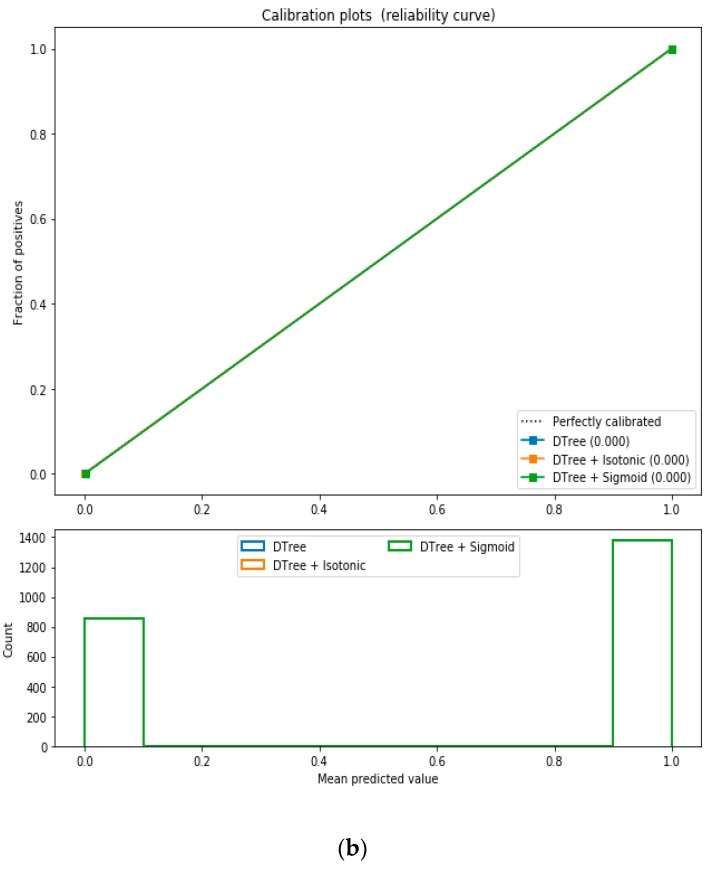
(**a**) Reliability curve to classify the “Eating-health-module” data with different ML classifiers. (**b**) Reliability curve to classify the “Eating-health-module” data with the “Calibrated Decision Tree”.

For calibration-based classification, we converted the multiclass “Eating-health-module” dataset to a binary classification problem following the logic that if (underweight, normal weight) then “body_composition” = 0, else 1. The uncalibrated “SVM”, “Decision Tree”, and “Random Forest” classifiers performed the best in the binary classification. On the contrary, the calibrated “DecisionTree” classifier outperformed the other classifiers, as depicted in Figure 14, following the “isotonic” and “sigmoid” calibration methods with a Brier score = 0.000. It illustrates that both the calibrated and uncalibrated “Decision Tree” classifiers give same performance.

The “Pima-Indians-diabetes-database” dataset has 768 records and nine features: “pregnancies”, “glucose”, “blood pressure”, “skin thickness”, “insulin”, “BMI”, “diabetes pedigree function”, “age”, and “outcome”. During the data pre-processing, we included records with age >20 and <60 and excluded features such as “pregnancies”, “insulin”, and “skin thickness”. 

**Figure 15:**
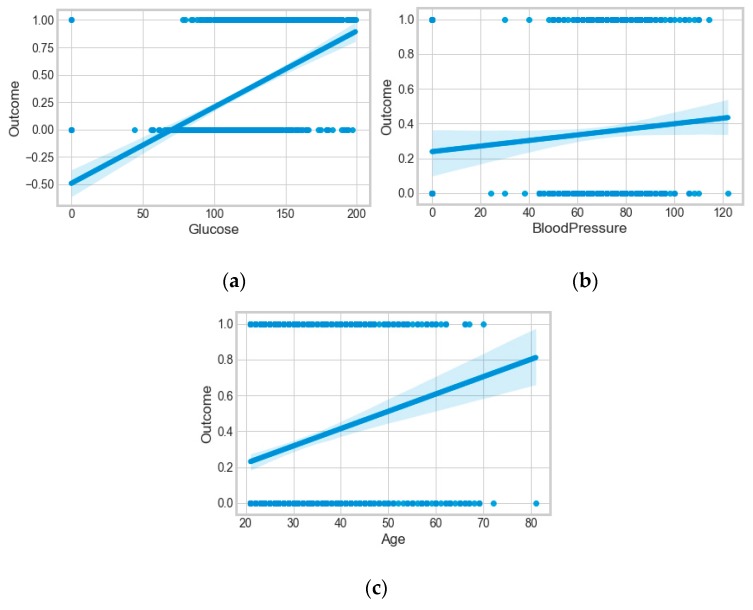
(**a**) Relationship of the outcome (obesity) with blood glucose; (**b**) relationship of the outcome (obesity) with blood pressure; (**c**) relationship of the outcome (obesity) with age.

**Figure 16:**
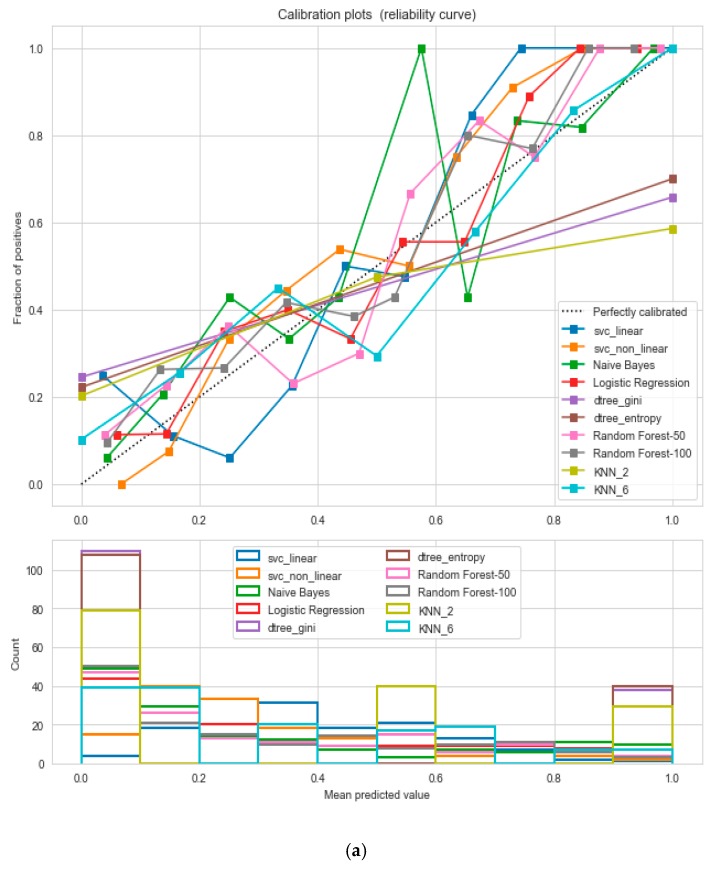

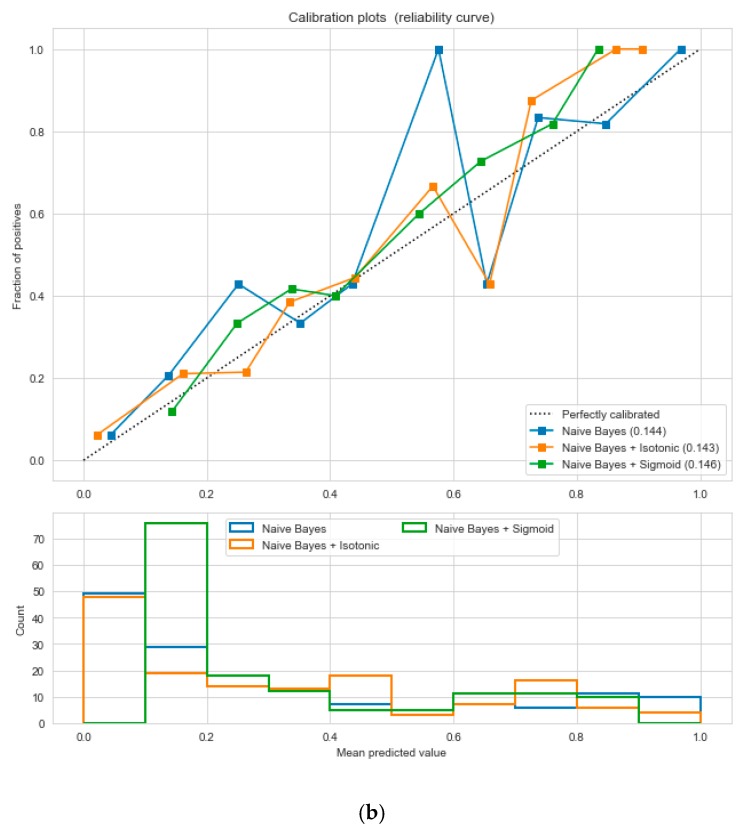
(**a**) Reliability curve to classify the “Diabetes” data with different ML classifiers. (**b**) Reliability curve to classify the “Diabetes” data with the “Calibrated LR”.

It resulted in 736 records. The regression analysis of the dataset resulted in a positive dependency in Figure 15, respectively. We used ML classification algorithms to classify the records among two classes, obese (0) and non-obese (1), under the feature column “outcome”. “GaussianNB”, “SVM”, and “Logistic Regression (LR)” outperformed the other classifiers, with a 5-fold cross-validation and 78% accuracy. The best parameters of “SVM” and “LR” were {‘C’: 10, ‘gamma’: 0.001, ‘kernel’: linear} and {’C’: 0.1, ’penalty’: ’l2’}, with a score of 0.78 following the grid search method. This analysis shows a relationship between obesity and diabetes. The logistic regression performed the best under the probability calibration method following the Brier score metric, as depicted in Figure 16.

The “cardiovascular-disease” dataset has 462 records and 22 features: “ind” (index), “sbp” (blood pressure), “tobacco”, “ldl” (lipid profile), “adiposity”, “famhist” (family history), “typea”, “obesity”, “alcohol”, “age”, and “chd” (cardiac disease). During the data pre-processing, we included records with age >20 and <60 and excluded features such as “ind” (index). 

**Figure 17:**
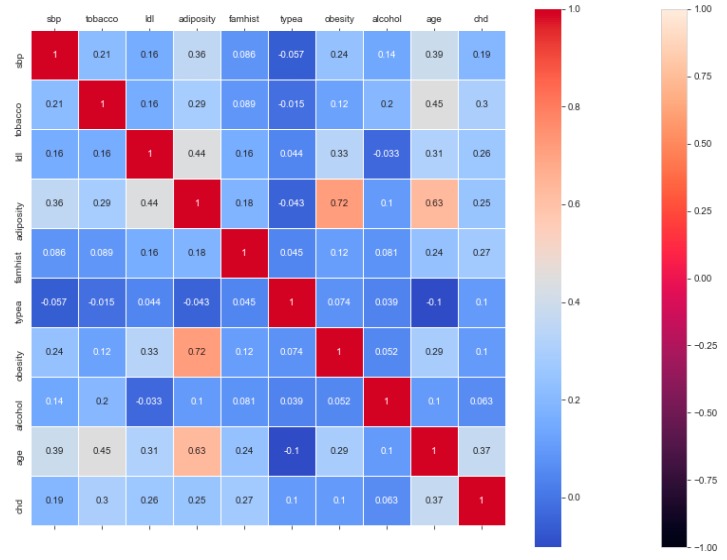
Correlation heatmap and classification accuracy of ML models to classify the “Cardiovascular-disease” data.

**Figure 18:**
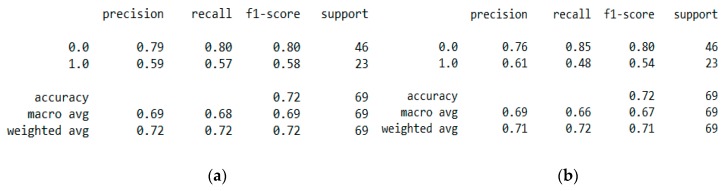
(**a**) Performance metric of the “SVM” classification with a 5-fold cross validation. (**b**) Performance metric of the “Logistic Regression” classification with a 5-fold cross validation.

This resulted in 342 records. The feature “chd” determines if a person has heart disease (1) or not (0). The regression analysis of the dataset revealed that blood pressure, tobacco consumption, lipid profile, adiposity, family history, obesity, drinking habit, and age has a strong connection with CVDs. In binary classification problems on the used heart dataset, SVM and logistic regression outperformed the other classifiers, with a 5-fold cross-validation and 72% accuracy. There is a a strong correlation between (a) “adiposity” and “obese” and (b) “age” and “adiposity”, with |r| = 0.72 and |r| = 0.63, respectively, as depicted in Figure 17. The SVM and logistic regression estimated the best parameters as {’C’: 10, ’gamma’: 0.001, ‘kernel’: linear} and {’C’: 7.74, ’penalty’: L2}, respectively, under grid search method; the corresponding performance metrics are as depicted in Figure 18. 

**Figure 19:**
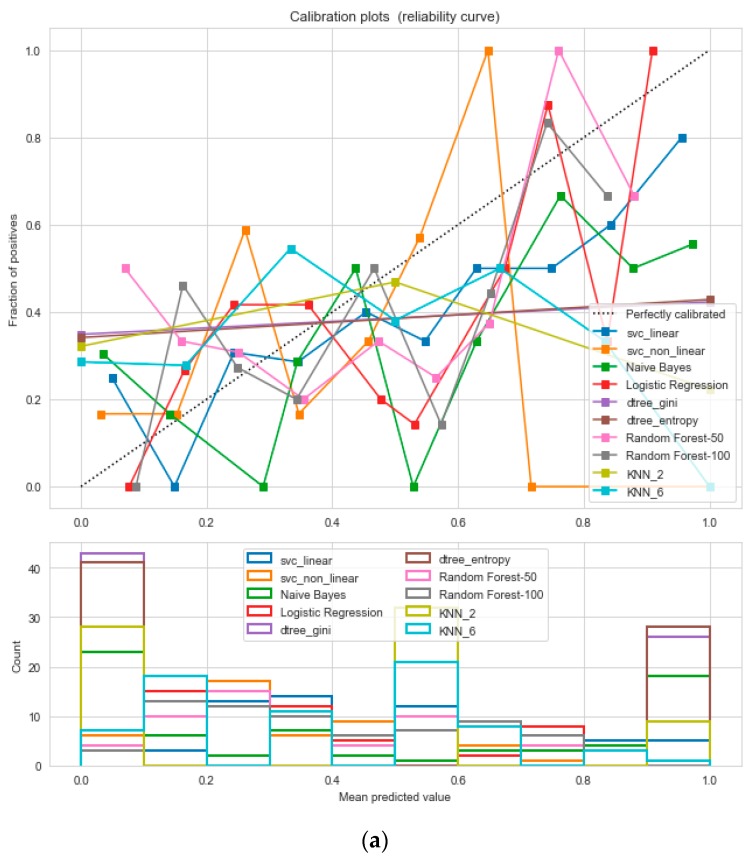

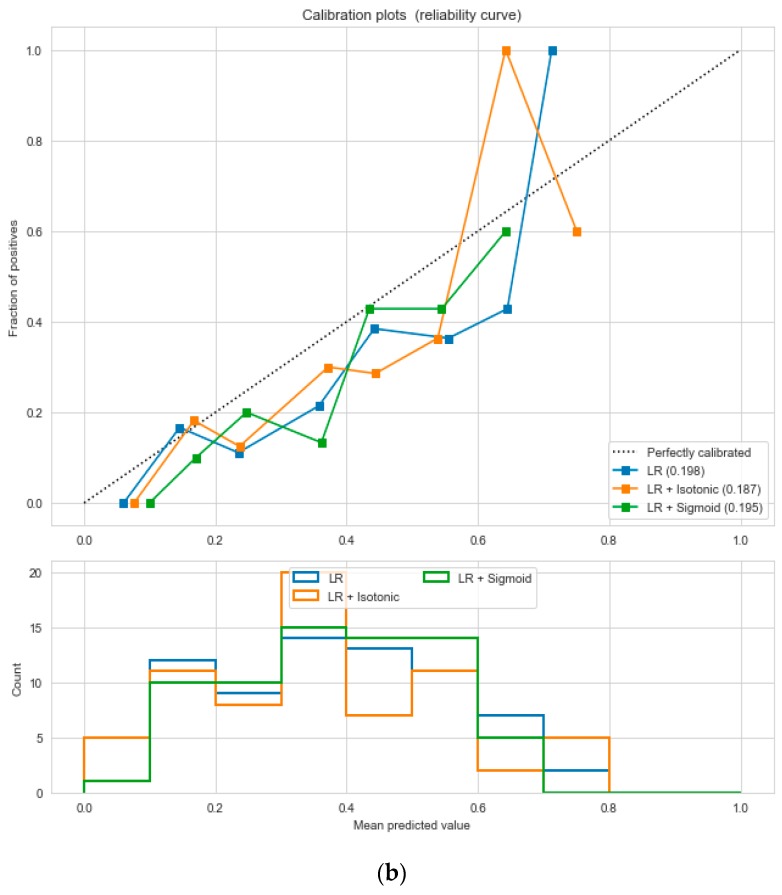
(**a**) Reliability curve to classify the “Cardiovascular disease” data with different ML classifiers. (**b**) Reliability curve to classify the “Cardiovascular disease” data with the “Calibrated LR”.

The logistic regression performed the best under the probability calibration method following the Brier score metric, as depicted in Figure 19. 

The synopsis of the above analyses has been outlined in Table 11 and Table 12. From the above data analyses, it is observed that with limited dataset SVM (linear kernel) and “Decision Tree” classifiers outperformed the other classifiers. The logistic regression returns well-calibrated predictions for the binary class classification problem, as it directly optimizes the log loss. The identified risk factors from the above analysis related to “obesity and overweight” can be summarized as: (a) BMI, (b) age, (c) tobacco consumption, (d) sweet beverages, (e) economic condition, (f) fast food, (g) sleeping pattern, (h) diet, (i) blood pressure, (j) blood glucose, (k) lipid profile, (l) adiposity, (m) exercise, and (n) family history. In brief, the identified core risk factors for obesity/overweight are nutritional conditions or curbing, education, socioeconomic conditions, dietary changes, and physical inactivity. The analyses have established our assumed hypothesis that obesity/overweight has a sharp relation with CVDs and diabetes type II, and some common risk factors are age, sex, cholesterol, lipid profile, sugar level, blood pressure, and family history.

## 6. Conclusions

From the review of different ML algorithms on publicly available health datasets in “Kaggle”, and “UCI”, we identified potential risk factors associated with obesity/overweight using statistical and machine learning and data visualization methods. We had trouble with the size of the datasets and the fact that they were not from same source. Thus, they represented different populations and integrating them together was difficult. However, their separate statistical analysis has given a good identification of the potential risk factors to be addressed and studied further relating to “obesity and overweight”. In the future, the multiclass classifiers could be extended with non-convex optimization (neural net) methods or ensembles. Our future study focuses on designing, developing, testing, and evaluating the performance of an intelligent eCoach system to achieve personal wellness goals, addressing obesity as a study case. An eCoach system can empower people to manage a healthy lifestyle with early risk predictions and appropriate individualized recommendations. Hence, the identification of an appropriate set of risk factors is necessary before real data collection for an eCoach system for our target population in the age group of >20 and <60, excluding pregnancy and genetic factors. The digital eCoaching system will capture data related to potential risk factors associated with “obesity/overweight” over time from an identified controlled trial in south Norway with both malea and females for a statistical analysis (regression and prediction). It will generate automated, personalized, contextual, and behavioral recommendations for its participants. 

## Figures and Tables

**Table 1:** Epidemiological study design [16].

Study Design	Type of Information Collected	Usage of the Information
Meta-analysis and systematic reviews	Summary of the evidence of predominance of obesity/overweight worldwide Summary of the evidence of physiological risks associated to obesity/overweight Summary of the evidence of risk factors associated to obesity/overweight Summary of the evidence of effectiveness of obesity/overweight prevention plan	Strategy and guideline planning
Qualitative and quantitative studies	Burden of obesity/overweight in society Correlation of risk factors with body energy imbalance Distribution of obesity prevalence among different age groups and socio-economic groups Identification of key risk factors, high risk groups of people, and related datasets Identification of used artificial intelligence (AI) models with their accuracy for classification and regression	Policy, algorithm selection, data selection, controlled trial selection, feasibility study, goal setting, planning, resource allocation, priority setting, impact analysis, and evaluation

**Table 2:** AI models and the risk factors related to obesity/overweight.

Researcher	Model Use	Risk Factors
DeGregory et al.	Linear and logistic regression, artificial neural networks, deep learning, decision tree analysis, cluster analysis, principal component analysis (PCA), network science, and topological data analysis	Inactivity, improper diet
Singh et al.	Multivariate regression methods and multilayer perceptron (MLP) feed-forward neural network models	BMI
Bassam et al.	Logistic regression, k-nearest neighbor (KNN), support vector machine (SVM)	Age, sex, body mass index (BMI), pre-existing hypertension, family history of hypertension, and diabetes (type II)
Meghana et al.	Automatic machine learning (AutoML)	cardiovascular diseases (CVDs)
Seyla et al.	SVM	Activity, nutrition
Jindal et al.	Random Forest	Age, height, weight, BMI
Zheng et al.	improved decision tree (IDT), KNN, artificial neural network (ANN)	Inactivity, improper diet
Dunstan et al.	SVM, Random Forest (RF), Extreme Gradient Boosting (XGB)	Unhealthy diet
Golino et al.	Classification tree, logistic regression	Blood Pressure (BP), BMI, Waist Circumference (WC), Hip Circumference (HC), Waist–Hip Ratio (WHR)
Pleuss et al.	Machine learning (ML) and 3D image processing	BMI, WC, HC
Maharana et al.	convolutional neural network (CNN)	Environment, context
Pouladzadeh et al.	CNN	Nutrition

**Table 3:** Selected datasets for the statistical analysis and machine learning.

Repository	Name	Source	Category
Kaggle	500_Person_Gender_Height_Weight_Index	www.github.com [35]	Obesity
Kaggle	Insurance	www.csueastbay.edu [36]	Obesity
Kaggle	Eating-health-module-dataset [37]	US Bureau of Labor Statistics	Obesity
Kaggle/UCI	Pima-Indians-diabetes-database	UCI Machine Learning	Diabetes
Kaggle	Cardiovascular-disease-dataset	Ryerson University	CVDs

**Table 4:** Short description of the selected datasets.

Type	Sample Size	Key Features
Person_Gender_Height_Weight_Index	500	Gender, height, weight
Insurance	1338	Age, sex, BMI, smoking, charge, location
Eating-health-module-dataset	11212	Sweet beverages, economic condition, fast food, sleeping, meat and milk consumption, drinking habit, exercise
Pima-Indians-diabetes-database	768	Blood glucose, blood pressure, insulin intake, and age
Cardiovascular-disease-dataset	462	Blood pressure, tobacco consumption, lipid profile, adiposity, family history, obesity, drinking habit, and age

**Table 5:** Python libraries for data processing [38].

No.	Libraries	Purpose
1	Pandas	Data importing, structuring, and analysis
2	NumPy	Computing with multidimensional array object
3	Matplotlib	Python 2-D plotting
4	SciPy	Statistical analysis
5	Seaborn, plotly	Plotting of high-level statistical graphs
6	Scikit-learn (Sklearn)	Machine learning, preprocessing, cross-validation, and evaluating the model’s performance
7	Graph Viz	Plotting of decision trees

**Table 6:** Statistical analysis methods on the selected datasets [32,39].

No.	Methods	Purpose
1	Mean, standard deviation, skewness	Distribution test
2	t-test, z-test, F-test, Chi-square	Hypothesis test
3	Shapiro–Wilk, D’Agostino’s K^2, and Anderson–Darling test	Normality test
4	Covariance, correlation	Association test
5	Histogram, Swarm, Violin, Bee Swarm, Joint, Box, Scatter	Distribution plot
6	Quantile analysis	Outlier detection

**Table 7:** Hypothesis testing methods.

Method	Description	Samples
T Test	Test if the mean of a normally distributed value is different from a specified value (µ0)	Sample size < 30
Z Test	Test if two samples are equal or not	Sample size > 30
ANOVA or F Test	Test multiple groups at the same time	More than 2 samples
Chi-Square Test	Check if observed patterns (O) of data fit some given distribution (E) or not.	Two categorical variables from a sample

**Table 8:** Statistical analysis methods on the selected datasets.

|r| Value	Meaning
0.00–0.2	Very weak
0.2–0.4	Weak to moderate
0.4–0.6	Medium to substantial
0.6–0.8	Very strong
0.8–1.0	Extremely strong

**Table 9:** Statistical analysis methods on the selected datasets [31,32,39].

Type	Name	Optimization Method
Classification	SVM (kernel = linear or rbf)	Gradient descent
Classification	Naïve Bayes	Gradient descent
Classification	Decision Tree (entropy or gini)	Information Gain, Gini
Classification	Logistic	Gradient descent
Classification	KNN	Gradient descent
Classification	Random Forest (RF)	Ensemble
Calibration Classification	Calibrated Classifier (CV)	Probability (sigmoid, isotonic)
Regression	Linear Regression	Gradient descent
Regression	KNeighbors Regressor	Gradient descent
Regression	Support Vector Regressor	Gradient descent
Regression	Decision Tree Regressor	Gain, Gini
Regression	Random Forest Regressor	Ensemble
Regression	Bayesian Regressor	Gradient descent
Regularization	Lasso (L1), Ridge (L2)	Gradient descent

**Table 10:** Machine learning model store [27].

Method	Implementation
Pickle string	Import pickle library
Pickled model	Import joblib from the sklearn.externals library

**Table 11:** The data processing synopsis with a discrimination analysis.

Name of the Dataset	Data Processing Reason	Best ML Model with Performance Metrics	Identified Risk Factors
Person_Gender_Height_Weight_Index	To check correlation between BMI and weight change. Comparing the performance of multiclass classifiers.	SVM classifier Accuracy: 95% Mean squared error (MSE): 0.08 Mean absolute error (MAE): 0.06 R^2^: 0.96	BMI
Insurance	To check the impact of identified health risk factors on weight change using regression and correlation. Comparing the performance of multiclass classifiers Comparing the performance of regression algorithms. To check if BMI has any relation with age or not.	Decision tree (DTree) classifier Accuracy: 99.64% MSE: 1.0 MAE: 0.0 R^2^: 1.0 RF regressor Accuracy: 82% MSE: 27240902.29 MAE: 3129.09 R^2^: 0.809	Age, sex, BMI, smoking habit, economic condition
Eating-health-module-dataset	To check the impact of the identified health risk factors on weight change using regression and correlation. Comparing the performance of multiclass classifiers.	DTree classifier Accuracy: 99.7% MSE:1.0 MAE: 0.0 R^2^: 1.0	Sweet beverages, economic condition, fast food, sleeping, meat and milk consumption, drinking habit, exercise
Pima-Indians-diabetes-database	To check the impact of the identified health risk factors on weight change using regression and correlation. Comparing the performance of multiclass classifiers. To check the relationship between diabetes type II and obesity.	SVM, Naïve Bayes, Logistic Regression (LR) Accuracy: 78% MSE: 0.209 MAE: 0.209 R^2^: 0.080	Blood glucose, blood pressure, and age
Cardiovascular-disease-dataset	To check the impact of the identified health risk factors on weight change using regression and correlation. Comparing the performance of multiclass classifiers. To check the relationship between heart disease and obesity.	SVM and Logistic regression Accuracy: 72% MSE: 0.275 MAE: 0.275 R^2^: −0.239	Blood pressure, tobacco consumption, lipid profile, adiposity, family history, obesity, drinking habit, and age

**Table 12:** The data processing synopsis with the calibrated classification.

Name of the Dataset	Best ML Model	Best Calibration Method	Uncalibrated Brier Score	Calibrated Brier Score
Person_Gender_Height_Weight_Index	Decision Tree	Isotonic	0.000	0.000
Insurance	Decision Tree	Isotonic	0.000	0.000
Eating-health-module-dataset	Decision Tree	Isotonic, Sigmoid	0.000	0.000
Pima-Indians-diabetes-database	Logistic Regression	Isotonic	0.144	0.143
Cardiovascular-disease-dataset	Logistic Regression	Isotonic	0.198	0.187

## Supplementary Materials

To reproduce the result, the codebase can be downloaded from GitHub repository: https://github.com/ayan1c2/Machine_Learning_In_Obesity.git.
